# The MalR type regulator AcrC is a transcriptional repressor of acarbose biosynthetic genes in *Actinoplanes* sp. SE50/110

**DOI:** 10.1186/s12864-017-3941-x

**Published:** 2017-07-25

**Authors:** Timo Wolf, Julian Droste, Tetiana Gren, Vera Ortseifen, Susanne Schneiker-Bekel, Till Zemke, Alfred Pühler, Jörn Kalinowski

**Affiliations:** 1 Microbial Genomics and Biotechnology, Center for Biotechnology, Bielefeld University, Universitätsstraße 27, 33615 Bielefeld, Germany; 2 Senior Research Group in Genome Research of Industrial Microorganisms, Center for Biotechnology, Bielefeld University, Universitätsstraße 27, 33615 Bielefeld, Germany; 3 Product Supply, Bayer Pharma AG, Friedrich Ebert Str. 217-475, 42117 Wuppertal, Germany

**Keywords:** *Actinoplanes*, Acarbose, MalR, AcrC, Transcriptional regulation, Actinomycetes

## Abstract

**Background:**

Acarbose is used in the treatment of diabetes mellitus type II and is produced by *Actinoplanes* sp. SE50/110. Although the biosynthesis of acarbose has been intensively studied, profound knowledge about transcription factors involved in acarbose biosynthesis and their binding sites has been missing until now. In contrast to acarbose biosynthetic gene clusters in *Streptomyces* spp., the corresponding gene cluster of *Actinoplanes* sp. SE50/110 lacks genes for transcriptional regulators.

**Results:**

The acarbose regulator C (AcrC) was identified through an in silico approach by aligning the LacI family regulators of acarbose biosynthetic gene clusters in *Streptomyces* spp. with the *Actinoplanes* sp. SE50/110 genome. The gene for *acrC*, located in a head-to-head arrangement with the maltose/maltodextrin ABC transporter *malEFG* operon, was deleted by introducing PCR targeting for *Actinoplanes* sp. SE50/110. Characterization was carried out through cultivation experiments, genome-wide microarray hybridizations, and RT-qPCR as well as electrophoretic mobility shift assays for the elucidation of binding motifs. The results show that AcrC binds to the intergenic region between *acbE* and *acbD* in *Actinoplanes* sp. SE50/110 and acts as a transcriptional repressor on these genes. The transcriptomic profile of the wild type was reconstituted through a complementation of the deleted *acrC* gene. Additionally, regulatory sequence motifs for the binding of AcrC were identified in the intergenic region of *acbE* and *acbD*. It was shown that AcrC expression influences acarbose formation in the early growth phase. Interestingly, AcrC does not regulate the *malEFG* operon.

**Conclusions:**

This study characterizes the first known transcription factor of the acarbose biosynthetic gene cluster in *Actinoplanes* sp. SE50/110. It therefore represents an important step for understanding the regulatory network of this organism. Based on this work, rational strain design for improving the biotechnological production of acarbose can now be implemented.

**Electronic supplementary material:**

The online version of this article (doi:10.1186/s12864-017-3941-x) contains supplementary material, which is available to authorized users.

## Background

Acarbose (acarviosyl-1,4-maltose) is used for the treatment of diabetes mellitus type II, as it supports the reduction of blood sugar levels, due to its inhibitory effect on alpha-glucosidases in the human intestine [1–3]. The Gram-positive actinobacterium *Actinoplanes* sp. SE50/110 is a natural producer of the pseudotetrasaccharide acarbose and the genome includes the acarbose biosynthetic (*acb*) gene cluster [4, 5]. Therefore, *Actinoplanes* sp. SE50 strains are used for the biotechnological production of acarbose [6]. *Actinoplanes* species are characterized by genomes with high G + C contents of 69–73%, can produce motile spores and typically grow in branched hyphae [7, 8].

Based on biochemical studies of the enzymes encoded by the *acb* gene cluster as well as genome-wide omics studies, models for the enzymatic pathways of acarbose biosynthesis have been proposed and targets for metabolic engineering have been suggested [3, 9–11]. However, functional studies concerning the acarbose biosynthesis based on genetic engineering of *Actinoplanes* sp. SE50/110 or rational strain designs have not been carried out until now. Recently, tools for genetic engineering of *Actinoplanes* sp. SE50 strains were developed [12, 13]. Combined with the high quality genome sequence and annotation of *Actinoplanes* sp. SE50/110 [14], targeted mutagenesis will facilitate the validation of acarbose biosynthesis and its regulation.

The transcriptional organization of the *acb* gene cluster, including transcription start sites, promoter elements and operon organization, was recently elucidated [14]. The cluster is divided into seven transcription units, with most of the genes coding for biosynthetic enzymes organized in one operon. The genes *acbZ*, *acbD* and *acbE* are transcribed monocistronically and encode proteins of the extracellular carbohydrate and acarbose metabolism. The genes *acbE* and *acbD* are located adjacently and oriented divergently [14]. However, profound knowledge about transcription factors involved in acarbose biosynthesis and their binding sites is missing until now. In contrast to acarbose biosynthetic gene clusters in *Streptomyces* spp. [15, 16], the *acb* gene cluster in *Actinoplanes* sp. SE50/110 lacks genes coding for transcription factors.

Nevertheless, it is known that expression of the genes *acbD* and *acbE* is inducible by maltotriose, when expressed heterologously in *Streptomyces lividans* [4]. It was suggested that dyadic symmetry element boxes (DSE) in the intergenic regions of the oppositely oriented genes *acbA* and *acbB* as well as *acbE* and *acbD,* might be possible operator sites for carbohydrate dependent transcriptional regulators [3]. Similar DSE boxes associated with maltose/maltotriose induction and glucose repression were identified upstream of alpha-amylase genes in several *Streptomyces* spp. [17, 18].

In this study, we expanded the toolbox for genetic engineering of *Actinoplanes* sp. SE50/110 through the successful application of PCR targeting (“ReDirect” technology), and applied this technology for the functional characterization of the MalR type transcription factor acarbose regulator C (AcrC). The rationale for classifying this transcription factor as a regulator of *acb* genes is shown by an in silico approach, cultivation experiments, transcriptomics as well as electrophoretic mobility shift assays for the elucidation of its DNA-binding motifs.

## Results

### In silico analysis for the identification of a transcriptional regulator of the acarbose biosynthetic gene cluster and construction of a deletion mutant

**Fig. 1:**
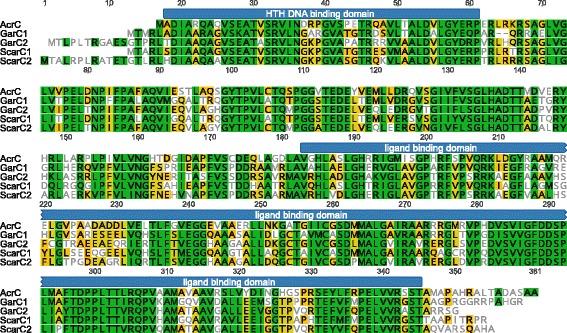
AcrC was identified through alignment with transcriptional regulators from acarbose biosynthetic gene clusters of streptomycetes. The protein alignment of AcrC from *Actinoplanes* sp. SE50/110, GarC1 and GarC2 from *S. glaucescens* as well as ScarC1 and ScarC2 from *S. coelicoflavus* is shown*.* The protein domains were determined with Pfam [62] and refer to the exact amino acid positions of AcrC. The alignment was performed with MUSCLE [21] in Geneious [63]

Recently, the transcriptional organization of the acarbose biosynthetic gene cluster (*acb* gene cluster), including transcription start sites, promoter elements and operon organization was elucidated [14]. However, profound knowledge about transcription factors involved in acarbose biosynthesis and their binding sites was missing until now. The *acb* gene cluster in *Actinoplanes* sp. SE50/110 lacks genes coding for transcriptional regulators. Interestingly, two other gene clusters for the production of acarviostatins have been identified in *Streptomyces* spp.. These are the *gac* gene cluster from *Streptomyces glaucescens* GLA.O [15, 19] and the *sct* gene cluster from *Streptomyces coelicoflavus* ZG0656 [16], which each include two LacI-type regulators (*garC1*, *garC2*, and *scarC1*, *scarC2*, respectively). When using protein alignment tools such as BLASTP [20] with the protein sequences of these regulators as an input and the *Actinoplanes* sp. SE50/110 genome for searching, the LacI family transcriptional regulator ACSP50_6387 was the best hit in all four cases. The pairwise identity of the regulators GarC1 and GarC2 from *S. glaucescens* GLA.O and ScarC1 as well as ScarC2 from *S. coelicoflavus* ZG0656 with ACSP50_6387 was between 59.7 and 63.4%, as determined through alignments using MUSCLE [21] (Fig. 1). These observations lead to the conclusion that ACSP50_6387 is a possible transcriptional regulator of the *acb* gene cluster. The *ACSP50_6387* gene was originally named *malR* and is located head to head to the maltose/maltodextrin ABC transporter gene cluster *malEFG* [11]. As this regulator also shows high similarities to MalR regulators, binding to the upstream region of the *malEFG* operon in other *Actinobacteria*, it was assumed that this regulator has a similar function in *Actinoplanes* sp. SE50/110 [22, 23]. In this study, it was shown that the LacI family regulator ACSP50_6387 is not the repressor of the *malEFG* operon, but is the first identified transcriptional regulator of the *acb* gene cluster, which is why it was named acarbose regulator C (AcrC). Conclusive evidence for this is given in the following.

A deletion mutant of the MalR-type regulator gene *acrC* was constructed using PCR targeting [24]. For this technology, also called “ReDirect” technology, a cosmid containing the chromosomal region surrounding *acrC* and the *malEFG* operon was modified by applying λ RED-mediated recombination [25]. The complete coding region of *acrC* was replaced with the selection marker *aac(3)IV*, conferring apramycin resistance and an *oriT* (RK2) for conjugational transfer of the cosmid. The gene disruption of *acrC* in *Actinoplanes* sp. SE50/110 was verified by PCR on isolated DNA and by sequencing of the PCR products. These results proved the successful application of the so-called “ReDirect” technology in *Actinoplanes* sp. SE50/110 for the first time.

### Establishment of whole genome microarrays for *Actinoplanes* sp. SE50/110 and application on a Δ*acrC* deletion mutant

In order to characterize the transcriptional regulator AcrC, comparative genome wide transcriptome analyses were conducted. Therefore, the wild type *Actinoplanes* sp. SE50/110 and the mutant Δ*acrC* were each cultivated in triplicates in minimal medium supplemented with maltose or glucose as single carbon source. Maltose minimal medium was used, as it is known as an acarbose production medium [26]. It was assumed that maltose or a metabolic product of maltose is an effector of AcrC, due to its similarity to MalR-like regulators. Therefore, maltose or a derivative might prevent the repressor AcrC from binding to its operator sites and consequently might lower the effect of a deletion mutant on the transcript levels of relevant genes. To better analyze the effect of the deletion mutant Δ*acrC* on the transcriptome, glucose minimal medium was used in parallel.

RNA samples from the biological replicates were taken in the middle of the growth phase of both strains in each maltose and glucose minimal medium, respectively. RNA was isolated and the three replicates were combined for each strain and condition. Subsequently, whole genome microarrays were used to identify genes regulated by AcrC. Agilent oligonucleotide microarrays were constructed, consisting of a total of 43,803 features and representing 8238 genes of *Actinoplanes* sp. SE50/110. Furthermore, the arrays contained 1417 control spots. The standard protocol for microarray hybridization was adapted due to the high G + C content of *Actinoplanes* sp. SE50/110. Additionally, the technical variance was determined in a “yellow experiment” (data not shown). The log_2_(fold change) cut-off (M-value) for a false discovery rate of 0.01 was determined as 1.1 and −1.1, respectively.

**Fig. 2:**
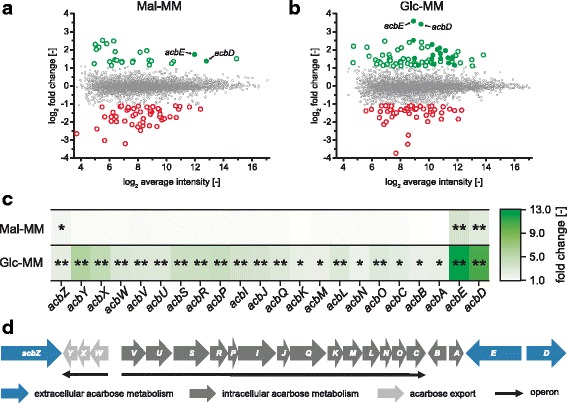
Differential transcriptional analysis of the deletion mutant Δ*acrC* compared to the wild type. **a** Ratio/intensity plot from whole genome microarrays of the strain *Actinoplanes* sp. SE50/110 Δ*acrC* compared to the *Actinoplanes* sp. SE50/110 wild type grown in maltose minimal medium (Mal-MM). Green and red dots represent genes with significantly different transcript levels in the Δ*acrC* strain. Filled dots show *acb* genes. **b** Ratio/intensity plot from whole genome microarrays of the strain Δ*acrC* compared to the wild type grown in glucose minimal medium (Glc-MM). **c** Heatmap of the fold change of transcript abundance for the genes of the *acb* gene cluster, derived from the microarray data shown in 2A and 2B. Significance of *p* < 0.05 is marked with a single asterisk, significance of *p* < 0.01 with two asterisks (t-test, two-sample, Holm). **d** Transcriptional organization of the *acb* gene cluster with protein localizations depicted by coloring

Whole transcriptome analysisallowed the identification of several genes for which different transcript abundances were measured when comparing the mutant Δ*acrC* with the *Actinoplanes* sp. SE50/110 wild type (Fig. 2). For each cultivation condition, the data from two arrays (dye swap) were combined to make statistically reliable conclusions. When using the RNA from the strains grown in maltose minimal medium, 23 genes with a log_2_(fold change) greater than 1.1 were determined indicating significantly higher transcript levels of these genes in the mutant (t-test *p* < 0.05). For 54 genes, an log_2_(fold change) less than −1.1 was determined and thus the transcript abundances were significantly lower in the mutant (t-test *p* < 0.05, Fig. 2a). In glucose minimal medium, the log_2_(fold change) was above 1.1 for 73 genes and below −1.1 (t-test *p* < 0.05) for 51 genes, when comparing the strain Δ*acrC* to the wild type (Fig. 2b). This data provides the first evidence for genes transcriptionally regulated by AcrC (full list of genes with significantly differential transcript abundancies in Additional file 1).

In total, significantly higher transcript amounts were detected for seven genes in the strain Δ*acrC* in both maltose and glucose minimal medium. Among them were uncharacterized (ACSP50_2985 and ACSP50_6701) and hypothetical proteins (ACSP50_6700), a predicted extracellular protein with unknown function (ACSP50_6253) and the gene *dapE2,* putatively coding for a succinyl-diaminopimelate desuccinylase. The *dapE2* gene is highly similar to the *dapE1* gene, but since the latter is located together with *dapC* in the *Actinoplanes* sp. SE 50/110 genome it is a possible paralog. *DapE2* is located downstream of *acrC*, which is why polar effects through the replacement of *acrC* with the highly transcribed apramycin resistance cassette cannot be ruled out. Apart from the gene *acrC* itself, only two additional genes were identified with significantly reduced transcript amounts in the Δ*acrC* strain in both maltose and glucose minimal medium. These included *ACSP50_2217*, coding for a NADPH:quinone reductase and *ACSP50_4307*, coding for an oxidoreductase.

Most striking when analyzing the genes with significantly different transcript amounts in both cultivation conditions, were two of the genes of the *acb* gene cluster. For *acbE* (fold change of 3.4 in maltose, 12.1 in glucose medium) and *acbD* (fold change of 2.6 in maltose, 10.7 in glucose medium) significantly elevated transcript levels were measured in the strain Δ*acrC* (Fig. 2). In glucose minimal medium, these represented the genes with the overall largest differences in the transcript amount. *acbE* and *acbD* are genes encoding proteins of the extracellular acarbose metabolism [27]. For the other *acb* genes, which code for proteins of the acarbose biosynthesis or the export of acarbose, no significant differences in RNA amounts were measured in maltose minimal medium. However, in glucose minimal medium an increased transcript level was detected for all *acb* genes in the transcription factor knockout strain (Fig. 2c). For *acbM*, *acbN* and *acbB*, the fold change was just below the cut-off of 2.1 (M-value 1.1) but above 1.9. For the remaining *acb* genes, the fold changes were between 2.2 and 5.7.

Strikingly, no significant differences in the transcript abundance for genes of the operon *malEFG* were measured with the microarrays. This is surprising, as the gene for AcrC is located in direct proximity to this operon on the opposite DNA strand. To validate this unexpected result, reverse transcription quantitative PCR (RT-qPCR) measurements were performed with RNA from cultivations in different carbon sources (data shown in Additional file 2). This way it was also possible to rule out that the lack of differences in the transcript levels for *malEFG* originate from maltose being the effector molecule and glucose acting through carbon catabolite repression. When comparing the strain Δ*acrC* with the wild type, no differences in the transcript amounts of *malE* could be detected with glucose, maltose, a mixture of glucose and maltose, glycerol, or mannitol as carbon source. However, with all tested carbon sources the transcript amounts of *acbE* were elevated in the Δ*acrC* strain compared to the wild type. The observations described here, are the first indications, that AcrC is a repressor of at least two *acb* genes and does not regulate the *malEFG* operon.

### The transcription of the genes *acbD* and *acbE* is regulated by the repressor AcrC

A complementation of *acrC* in the deletion mutant Δ*acrC* was conducted to rule out polar effects of the gene replacement and to prove that the transcriptomic as well as phenotypic effects of the Δ*acrC* mutant can be attributed to the repressor effects of the transcriptional regulator. For the complementation of *acrC* in the deletion mutant Δ*acrC*, the φC31-based integrative vector pSET152 was used, for which the integration site in *Actinoplanes* sp. SE50/110 is known [12].

**Fig. 3:**
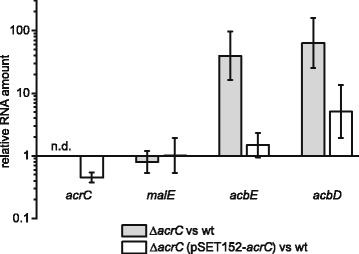
Relative RNA amounts of single genes in the deletion and complementation strain compared to the wild type. Relative transcript abundances of the deletion strain *Actinoplanes* sp. SE50/110 Δ*acrC* and the complementation strain *Actinoplanes* sp. SE50/110 Δ*acrC* (pSET152-*acrC*) were compared with the wild type *Actinoplanes* sp. SE50/110 (wt). The means and standard derivations of three biological replicates are shown. RNA was isolated from the growth phase of shake flask cultivations in glucose minimal medium and analyzed by RT-qPCR

The complementation of *acrC* and the effect on the transcription of the genes *malE, acbD* and *acbE* was analyzed through RT-qPCR (Fig. 3). Therefore, RNA isolated from the middle of the growth phase of strains grown in glucose minimal medium was used. The transcript levels of the single genes in the Δ*acrC* deletion strain as well as the complementation strain, were compared to the levels of the wild type. The complementation of *acrC* was validated, as only a slightly reduced relative transcript amount compared to the wild type was measured (fold change 0.45), but no transcripts were detected in the Δ*acrC* deletion strain. The results of the RT-qPCR analysis for the *malE* gene are in line with the data from the microarray, confirming that the transcription of *malE* is not influenced by AcrC. The relative transcript amounts for the genes *acbD* and *acbE* in the deletion strain Δ*acrC* were significantly elevated compared to the wild type strain and therefore validated the results of the microarrays (fold change 39.5 for *acbE* and 63.3 for *acbD*). In the complementation strain, the transcript amounts for these genes were only moderately elevated, showing the nearly successful reconstitution of the transcriptomic profile of the wild type (fold change 1.5 for *acbE* and 5.1 for *acbD*). It should be noted that the transcription of the genes *acbD* and *acbE* is highly regulated during growth and dependent on the growth phase of *Actinoplanes* sp. SE50/110 (our unpublished results). This can have a strong impact on the variance of biological replicates.

### AcrC has an effect on the acarbose production

**Fig. 4:**
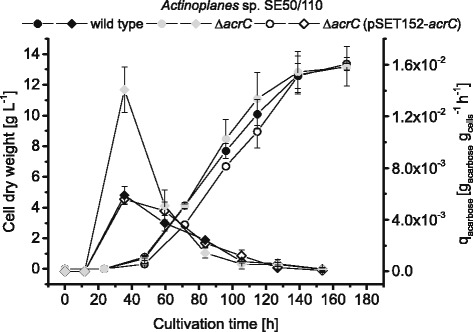
Growth and product formation of the wild type, deletion and complementation stain. Cell dry weight (*circles*) and specific product formation rates (*q*_*Acarbose*_ *, diamonds*) of the *Actinoplanes* sp. SE50/110 wild type, the deletion strain Δ*acrC* and the complementation Δ*acrC* (pSET152-*acrC*). Samples were taken from shake flask cultivation in maltose minimal medium inoculated with spores. The means and standard derivations of five biological and two technical replicates are shown

Comparative cultivations of the *Actinoplanes* sp. SE50/110 wild type, the mutant Δ*acrC* and the complementation strain Δ*acrC* (pSET152-*acrC*) were carried out to examine differences in growth and acarbose production. When comparing the three strains with respect to the cell dry weight, no significant differences were detected in growth behavior (Fig. 4). The production of different acarviose metabolites by *Actinoplanes* sp. SE50/110 is dependent on the available carbon source. When supplying glucose as carbon source, mainly acarviosyl-glucose is formed, which is why no production of acarbose is expected under these conditions [26]. Therefore, the acarbose concentration was determined solely for the cultivation in minimal medium with maltose, since acarviosyl-maltose (acarbose) is formed under these conditions [6, 26].

For cultivations of *Actinoplanes* sp. SE50/110 in maltose minimal medium in shake flasks, an acarbose concentration of up to 0.98 g L^−1^ for the wild type, 0.93 g L^−1^ for the deletion mutant and 0.75 g L^−1^ for the complementation strain was achieved. This corresponds to the expected product titer between 0.7 g L^−1^ and 1.0 g L^−1^ described in the literature for these conditions [3, 26]. In the early growth phase of the cultivations, a maximum of the specific product formation rate was obtained for all strains (Fig. 4). This shows that acarbose is produced during growth and not in the stationary phase and confirms the hypothesis of biomass-associated acarbose production of *Actinoplanes* sp. SE50/110 [26]. However, the strains differed with respect to the specific product formation level, defined as produced acarbose normalized to the mean cell dry weight and cultivation time. A significantly higher maximal specific product formation rate was achieved after 47.5 h in the *ΔacrC* strain (1.4 × 10^−2^ ± 0.2 × 10^−2^ h^−1^) compared to the wild type (5.9 × 10^−3^ ± 0.7 × 10^−3^ h^−1^) and the complementation strain (5.5 × 10^−3^ ± 0.4 × 10^−3^ h^−1^). Thus, there is an effect of AcrC expression on the product formation of acarbose in the early growth phase.

### The intergenic region between *acbE* and *acbD* features a binding site for AcrC

For the identification of precise binding sites of AcrC, band shift assays were carried out. Therefore, the AcrC protein was expressed in *Streptomyces lividans* TK23 and purified through a C-terminal hexa-histidine tag. The successful expression and purification of AcrC-His_6_ was verified by SDS page and a tryptic peptide fingerprint analysis using MALDI-ToF-MS/MS (Data not shown). Electrophoretic mobility shift assays (EMSA) were carried out with the purified protein and Cy3 labeled PCR fragments.

**Fig. 5:**
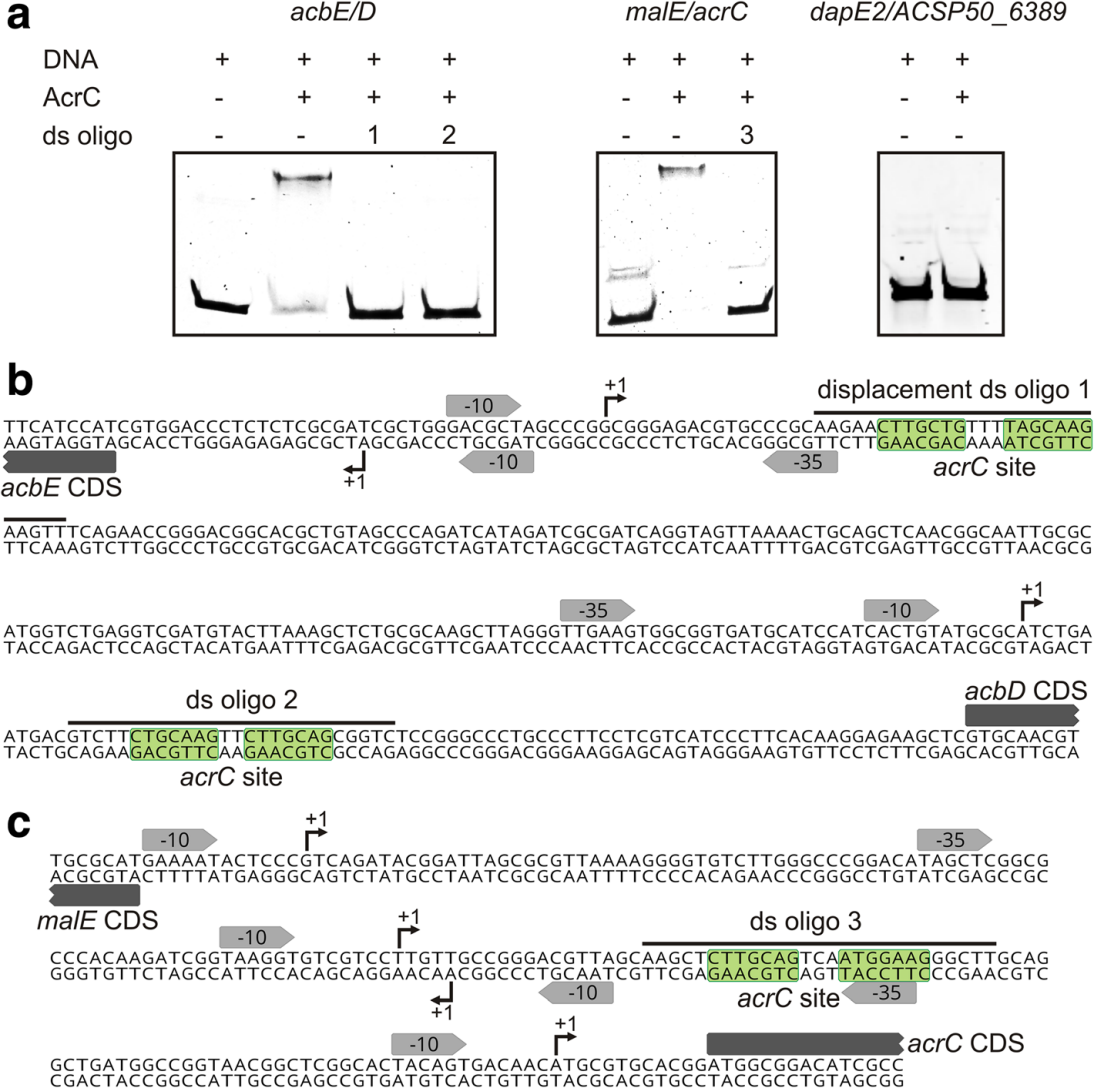
Electrophoretic mobility shift assays with AcrC protein and the intergenic region of *acbE* and *acbD*. **a** EMSAs with the 342 bp fragment of the intergenic region of *acbE*/*acbD*, the 217 bp intergenic region *malE*/*acrC* as well as the 203 bp region *dapE/ACSP50_6389*. 0.05 pmol Cy3 labeled PCR fragments were incubated with 80 pmol purified AcrC protein, 0.05 μg herring sperm DNA for blocking of unspecific binding, and 100 mg BSA. 12.5 pmol unlabeled double-stranded oligonucleotides (ds oligo) covering the *acrC* site plus 5 bp up- and downstream were added as indicated. Separation was carried out with 10% native polyacrylamide (TBE) gels and visualized by fluorescence imaging. **b** Intergenic region of *acbE* and *acbD* used for the EMSAs with the promoter motives described in [14] and the *acrC* binding sites. **c** Intergenic region of *malE* and *acrC* used for the EMSA with promoter motives

When using the intergenic region of *acbE* and *acbD* as well as the intergenic region of *malE*/*acrC* together with AcrC, a retardation of the DNA was observed. No bandshift was detected when using the upstream region of *dapE2* as a control (Fig. 5a). Therefore, AcrC binds to the promoter regions of *acbE* and *acbD* as well as of *acrC* itself but not to the promoter region of *dapE2*.

An analysis of the intergenic region of *acbD* and *acbE* revealed two potential DNA binding sites with inverted repeat sequences, which are typical for the specific binding of transcriptional regulators [28, 29]. Upstream of the translation start of *acbE*, the motif 5′-CTTGCTG-3 bp-TAGCAAG-3′ (O1) is found at a distance of 60 bp. The TSS of *acbE* is located 40 bp downstream of this palindromic motif. A secondary TSS of *acbD* is located 21 bp upstream of this motif. Upstream of the start codon of *acbD* (50 bp) the motif 5′-CTGCAAG-2 bp-CTTGCAG-3′ (O2) can be identified. The primary TSS of *acbD* can be found 15 bp upstream of this motif (Fig. 5b). A similar inverted repeat motif is also located in the intergenic region of *malE* and *acrC,* but with a weaker consensus sequence in the second repeat of 5′-CTTGCAG-3 bp -ATGGAAG-3′. The repeat is found downstream of two *acrC* TSS as well as upstream of one *malE* and a third *acrC* TSS (Fig. 5c). When unlabeled double-stranded oligonucleotides covering only these motifs were added to the EMSAs as competitive DNA in excess amounts, the binding of AcrC to the DNA was reversed (Fig. 5a). A complete displacement was observed starting at a 50 fold molar excess of the double-stranded displacement oligonucleotide over the labeled PCR fragment. When using a 25 fold excess, the displacement was partial (data not shown). This is a proof that the identified DNA regions are required AcrC binding. The identified motifs were used to build a position weight matrix and the *Actinoplanes* sp. SE50/110 genome was scanned for additional motifs. However, the motif was not identified upstream of other genes with significantly different transcript amounts when comparing the Δ*acrC* with the wild type through microarrays.

To identify a possible effector of AcrC, which interacts with the ligand-binding domain and causes its dissociation from the DNA-binding site by a conformational change, different sugars were added to the protein-DNA mix. However, a retardation of the DNA was still observed, when adding glucose, galactose, maltose, maltotriose or acarbose in a range of 1 to 20 mM (data not shown). Therefore, an effector could not be identified yet.

## Discussion

### Genetic engineering technologies and whole genome microarrays were established to characterize the transcription factor AcrC

The transcription factor AcrC was identified through an in silico approach by comparing the regulators of the acarbose biosynthetic gene clusters from *S. glaucescens* GLA.O [15] and *S. coelicoflavus* ZG0656 [16] with the genome of *Actinoplanes* sp. SE50/110. AcrC is a member of the LacI/GalR family of transcriptional regulators, which is mainly composed of repressor proteins of genes involved in carbohydrate and nucleotide metabolism [30, 31].

After the in silico identification of AcrC as a possible transcription factor of the *acb* gene cluster, methods for creating deletion mutants as well as a cost effective genome wide transcriptomics method with a relatively fast data evaluation pipeline were needed. When the work on AcrC was conducted, both elements were missing for *Actinoplanes* sp. SE50/110, and therefore PCR targeting (“ReDirect” technology) and genome wide microarrays were established for this organism.

The gene of *acrC* was replaced with an apramycin resistance cassette by applying PCR targeting [24], which proved the successful application of this technology in *Actinoplanes* sp. SE50/110 for the first time. This expands the toolbox for genetic engineering of *Actinoplanes* sp. SE50/110 additionally to the application of integrative vectors [12] and the meanwhile adapted genome editing using CRISPR/Cas9 [13].

The applicatiin of microarrays and RT-qPCR showned that the transcript levels of the genes *acbE* and *acbD* were elevated in the Δ*acrC* strain. This effect, caused by the deletion of *acrC,* was reversed by a complementation of *acrC*, confirming the successful reconstitution of the transcriptomic profile of the wild type. Although a clear effect of the complementation was shown, the transcript amount of *acrC* was only half as large as the transcript amount of the wild type, possibly resulting in slightly increased transcript amounts for *acbE* and *acbD* in the complementation strain compared to the wild type. An explanation for this could be possible polar effects at the integration site on the transcription of *acrC*. Another reason for the slight variances between the transcript levels of the wild type and Δ*acrC* strain could be that the transcription of the genes *acbD* and *acbE* is highly regulated during growth and dependent on the growth phase of *Actinoplanes* sp. SE50/110. This can lead to variances on the transcript levels between the strains, as it was observed for the comparison of the relative RNA amount of *acbD* in the complementation strain with the wild type.

Polar effects on neighboring genes were also observed through the replacement of *acrC* with the highly transcribed antibiotic resistance cassette. The gene *dapE2*, located directly downstream of *acrC*, is transcribed stronger in the strain Δ*acrC* and this effect was not reversed through the complementation (data not shown). Additionally it was shown that AcrC does not bind to the upstream region of *dapE2*, leading to the conclusion that the increased transcription of this gene in the mutant strain is caused by polar effects. Such effects on neighboring genes are unavoidable when applying PCR-targeting by replacing a target gene with a resistance marker cassette [32, 33]. This method can be expanded and improved by removing the antibiotic resistance cassette through site-specific recombination systems [34, 35], but this has not yet been applied to *Actinoplanes* sp. SE50/110. Alternatively, the recently established CRISPR/Cas9 technology enables scar-free and resistance marker-free deletions in the genome of *Actinoplanes* sp. SE50/110 with a single conjugation [13].

Well-functioning and reliable genetic engineering technologies in combination with fast and easily applicable whole genome transcriptomic methods will be indispensable for the clarification of regulatory networks in *Actinoplanes* sp. SE50/110. Although RNA-Seq has several advantages over microarrays, such as its single-nucleotide resolution and a much greater (log-linear) dynamic range [36, 37], the latter still have a legitimacy, as they can be used to simultaneously screen multiple samples in a cost-effective manner. The genome of *Actinoplanes* sp. SE50/110 harbors about 500 genes, which contain predicted DNA binding domains and might function as transcriptional regulators, of which now only the first one is functionally characterized. The methods established here will be helpful for the screening of many more transcription factors and understanding their biological functions. This knowledge will be of high value for metabolic engineering of this biotechnologically important organism.

### AcrC is the missing repressor of the acarbose biosynthetic gene cluster

When comparing the whole transcriptome of the deletion mutant Δ*acrC* with the *Actinoplanes* sp. SE50/110 wild type, it was noticeable that no significant differences in the transcript abundance for genes of the maltose/maltodextrin ABC transporter operon *malEFG* were detected. This was not expected, as the gene coding for AcrC is located adjacently and divergently oriented to *malEFG*. Furthermore, its function was predicted as a MalR-type regulator and AcrC shows high similarities to MalR regulators, acting as a repressor of the *malEFG* operon in other *Actinobacteria* [22, 23]. The deletion of *malR* in *S. coelicolor* results in a glucose-insensitive transcription of *malE* [22, 38]. The transcriptional repressor MalR from *S. lividans* was shown to not only bind to regulatory sequences upstream of *malEFG*, but also to operator sites upstream of alpha-amylase genes [23, 39, 40]. Glucose repression of alpha-amylase genes mediated through LacI/GalR type transcription factors was also reported for other Gram-positive bacteria [41–43]. In this study, it was shown that AcrC binds to the intergenic region of *acbE* and *acbD* in *Actinoplanes* sp. SE50/110 and acts as a transcriptional repressor on these genes. AcbE is an acarbose-resistant alpha-amylase, which degrades starch and maltodextrins to maltose and maltotriose or higher malto-oligosaccharides [44]. The gene *acbD* encodes an acarviose transferase, which is proposed to catalyze the transfer of acarviosyl moieties from acarbose to the hydroxyl group of various sugars [45, 46]. The architecture of the catalytic site of AcbD is similar to other enzymes of the alpha-amylase family [44, 46]. Although the MalR type regulator AcrC of *Actinoplanes* sp. SE50/110 does not influence the transcription of the *malEFG* operon, it still binds upstream of similar genes as MalR does in *Streptomyces* spp.

Two binding sites for AcrC, each composed of a palindromic 7 bp repeat (5′-CTTGC(A/T)G-3′) where identified in the intergenic region of *acbE* and *acbD*. The regulatory motif resembles the core binding site of MalR in *S. lividans*, which is described as 5′-CTTGCAG-3′, occurring as an inverted and a direct repeat upstream of *malE* but downstream of the promoter site [23]. Additional motifs were identified upstream of amylase and chitinase genes as direct or inverted repeats with a spacer of 3–15 bp [40]. In *Actinoplanes* sp. SE50/110 one of the operator sites is located downstream of the *acbD* transcription start sites and therefore blocks the RNA polymerase, but the other operator is located upstream of the promoter of *acbE*. However, the binding motif is located three base pairs upstream of the −35 region of this promoter, possibly acting by sterically blocking the RNA-polymerase from binding to the promoter. The close proximity of the two operator sites (182 bp) hints towards a possible tetrameric protein assembled of two homodimers, similar to the *E. coli* lactose repressor protein LacI [47, 48]. The repressor function of LacI is strengthened by DNA-looping with two operator sequences [49–51]. AcrC could form a similar structure, causing nearly the complete intergenic region between *acbE* and *acbD* to from a loop, thereby blocking all three promoters and increasing the repression effect. In *Actinoplanes* sp. SE50/110 the consensus-binding motif for AcrC also occurs as an inverted repeat with in the intergenic region between *malE* and *acrC.* Binding of AcrC to this region was shown with band shift assays. However, the potential binding site is located upstream of the *malE* TSS and downstream of two out of three *acrC* TSS. Together, with the observation that the transcription of *malE* is unchanged when deleting *acrC*, it can be assumed that only a transcriptional autoregulation of *acrC* occurs.

The consensus binding motif of AcrC was not identified upstream of the six additional genes with significantly different transcript amounts in both carbon source conditions. Although not consistently differentially transcribed in both conditions, transcriptional regulators were among the genes with significantly different transcript amounts in each condition. Therefore, indirect effects through changed metabolite concentrations or affected regulatory networks cannot be ruled out as cause for the differential transcript levels of these genes.

An effector molecule interacting with the ligand-binding domain of AcrC and thereby leading to a detachment of the repressor from the operator site was not detected through in vitro band shift assays. Nevertheless, the effect of the *acrC* deletion on the transcription of the *acb* genes, in particular *acbE* and *acbD*, was stronger in glucose containing medium compared to maltose minimal medium. This could indicate a detached repressor from the operator in maltose conditions. Combined, this could lead to the conclusion that maltose itself is not the effector of AcrC but a metabolic product directly derived from it. Maltodextrins can be built up intracellularly from maltose [52, 53] and are therefore promising candidates to be the effectors of AcrC, as it was also shown for MalR in *S. lividans* [23].

During the early growth phase, a significantly higher maximal specific product formation rate was achieved in the *ΔacrC* strain compared to the wild type and the complementation strain. Thus, there is a clear effect of AcrC expression on the acarbose formation in the early growth phase. This supports the assumption that AcrC is responsible for the repression of genes of the *acb* gene cluster in vivo, as the acarbose production is directly influenced by the deletion of the transcriptional regulator *acrC*. Based on literature and current models, acarbose is formed intracellularly and the extracellular proteins AcbE and AcbD are not directly involved in acarbose biosynthesis, when growing *Actinoplanes* sp. SE50/110 in maltose minimal medium [3, 26]. Therefore, a direct correlation of the transcription of the genes *acbE* and *acbD* with acarbose formation is not expected. It could be suspected that the gene products of *acbD* and *acbE* have additional enzymatic functions or that indirect effects such as feedback inhibition might influence acarbose formation.

The ABC transporter MalEFG was suggested as a possible acarbose-metabolite re-importer and AglEFG might be an additional maltose/maltodextrin importer [10, 54]. The proteins MalE, MalF and MalG were detected in high abundancies in both maltose and glucose-grown *Actinoplanes* sp. SE50/110 cultures [11]. This could lead to the conclusion that MalEFG imports acarviosyl metabolites independently from the available carbon source and could explain a possible evolutionary change of the AcrC regulon in *Actinoplanes* sp. SE50/110, dependent on the presence of the functional acarbose cluster. However, it could be beneficial to regulate the energy consuming expression and secretion of AcbE and AcbD, depending on the available carbon source. This function is implemented by AcrC in *Actinoplanes* sp. SE50/110 and could explain the special interaction between AcrC and the transcription of *acb* genes.

## Conclusions

The identification of AcrC as a repressor of genes of the acarbose biosynthetic gene cluster is an important step towards understanding the transcriptional regulation of the acarbose biosynthesis. This study not only describes the first documented transcription factor of the *acb* gene cluster in *Actinoplanes* sp. SE50/110 but is also the first functional study of genetic engineering that influences acarbose production in the biotechnologically important rare actinomycete *Actinoplanes* sp. SE50/110. Genetic engineering technologies were developed and can be used in combination with the described microarrays as well as RNA-Seq, to further elucidate the complex regulatory network of *Actinoplanes* sp. SE50/110. Based on this work, rational strain design for the improvement of acarbose production can be carried out.

## Methods

### Strains, media and reagents

All standard cloning procedures were carried out with *Escherichia coli* DH5αMCR [55]. *E. coli* BW25113/pIJ790 [24] was used for λ RED recombineering of cosmids. *E. coli* ET12567/pUZ8002 [56] was used as a conjugation host for the target organism *Actinoplanes* sp.SE50/110 (ATCC 31044) to generate mutant strains (this study). *Streptomyces lividans* TK23 [57] was used for overexpression of AcrC.

*Actinoplanes* sp. SE50/110 was grown on soy flour medium agar (SFM; 20 g L^−1^ soy flour, 20 g L^−1^ mannitol, 20 g L^−1^ agar, pH 8, tap water) and in NBS medium for molecular cloning procedures as well as strain maintenance. Minimal medium was supplemented with 2.4 C-mole of the respective sugar as carbon source. The composition of the liquid media is described elsewhere [10]. When needed, chloramphenicol (25 μg mL^-1^), kanamycin (50 μg mL^-1^), apramycin (50 μg mL^-1^) or hygromycin (100 μg/mL) was added to the media.

Soy flour (full fat) was used from Sobo Naturkost (Cologne, Germany) and purchased at a local store. For all PCRs, Phusion High-Fidelity PCR Master Mix with GC Buffer (NEB, Ipswich, MA, USA) was used. Gibson assembly master mix was prepared with Phusion High-Fidelity DNA Polymerase (Thermo Fisher Scientific, Waltham, MA, USA), T5 Exonuclease (Epicentre, Madison, WI, USA) and TaqDNA Ligase (NEB, Ipswich, MA, USA).

### Cultivation of *Actinoplanes* sp. SE50/110 and quantification of acarbose

For the cultivation of *Actinoplanes* sp. SE50/110 50 mL of medium were inoculated with 1 mL of spore suspension. Spores were harvested from freshly grown SFM agar plates with cultures grown for 6–7 days at 28 °C after uniformly plating 300 μL of a glycerol stock. Spores were washed off by adding 2 mL ddH_2_0 and carefully detaching them with a cotton swab. One plate resulted in roughly 1 mL spore suspension. The suspension of all plates for one strain was mixed before inoculation.

Cell dry weights were determined by harvesting 1 mL of cell suspension in weighed reaction tubes (20,000 *g*, 5 min). The supernatant was stored at −20 °C for acarbose quantification. The cell pellets were washed twice with ddH_2_0, dried at 70 °C for 48 h and weighed. For subsequent RNA isolation, 1 mL of cell suspension was centrifuged for 15 s at 16,000 *g* and immediately frozen in liquid nitrogen. Cell pellets were stored at −80 °C until further processed for RNA isolation.

Acarbose in the supernatant of *Actinoplanes* sp. SE50/110 cultivations was quantified by HPLC. Therefore, the supernatant was centrifuged (20,000 *g*, 2 min) to remove residual particles. Afterwards, 200 μL supernatant were mixed with 800 μL methanol, vortexed and centrifuged again (20,000 *g*, 2 min) to remove the resulting precipitate. The supernatant was transferred to HPLC vials and analyzed in a HPLC system (Finnigan Mat P4000 pump, AS3000 autosampler and UV6000LP detector, Thermo Fisher Scientific, Waltham, MA, USA). A flow of 1 mL min^−1^ of a mixture of 68% acetonitrile and 32% phosphate buffer (0.62 g L^−1^ KH_2_PO_4_ and 0.38 g L^−1^ K_2_HPO_4_·2H_2_O) was applied on a Hypersil APS-2 amino LC column (125 × 4 mm and 3 μm particle size, Thermo Fisher Scientific, Waltham, MA, USA) heated to 40 °C. The detection of acarbose was carried out with an UV detector at 210 nm. The acarbose concentration calculated with from the peak area and with a calibration curve.

### Construction of *Actinoplanes sp.* SE50/110 mutants

The regulator gene *acrC* was disrupted from start to stop codon in *Actinoplanes* sp. SE50/110 by applying PCR targeting, also called ReDirect. The ReDirect protocol (version 1.4) was carried out as described in detail elsewhere [25]. All primers used in this study are listed in Additional file 3. The plasmid pIJ773 [24] (received from B. Ostash, Ivan Franko National University of Lviv, Ukraine was used as template for the disruption cassette containing an apramycin resistance (*aac(3)IV*) and an *oriT* (RK2). The chromosomal sequence of *acrC* on a pcc2FOS based fosmid, containing the genomic region 12,914 bp downstream to 24,255 bp upstream of *acrC,* was replaced with the disruption cassette. The chloramphenicol resistance cassette on the pcc2FOS vector was replaced by a hygromycin resistance gene (received from L. Horbal, Helmholtz Institute for Pharmaceutical Research Saarland (HIPS), Germany) as a second selection marker. Conjugation of the cosmid was carried out as described previously [12]. After purification of exconjugants from *E. coli*, successful double-crossovers were verified by apramycin resistance and recovery of hygromycin sensitivity.

In order to complement the disrupted gene in the Δ*acrC* strain, a modified version of the integrative vector pSET152 [58] was used. The apramycin resistance gene of pSET152 was exchanged for a hygromycin resistance gene and the *acrC* gene including the 5′-UTR and promoter region (determined with data from [14]) was cloned in the multiple cloning site by isothermal Gibson assembly [59].

DNA of *Actinoplanes* sp. SE50/110 strains was isolated as described before [13]. PCR was used to confirm the constructed cosmids and plasmids as well as the genotype of all *Actinoplanes* sp. SE50/110 strains. PCR fragments were purified and Sanger sequencing was carried out by the in-house sequencing core facility.

### Transcriptomic analyses

#### RNA isolation

For RNA isolation frozen cell pellets were suspended in 800 μL RLT buffer (RNeasy mini kit, Qiagen, Hilden, Germany) and transferred to 2 mL lysing matrix tubes (0.1 mm spherical silica beads, MP Biomedicals, Santa Ana, California, USA). Cell disruption was carried out in a homogenizer (FastPrep FP120, Thermo Fisher Scientific, Waltham, MA, USA) for two times 20 s at speed setting 6.5 and 1 min on ice in between. Subsequently, the cell suspension was centrifuged for 3 min at 13,000 *g* and 4 °C. The supernatant was used for RNA extraction using a Qiagen RNeasy mini kit in combination with an RNase-free DNase kit (Qiagen, Hilden, Germany) for on-column and off-column DNA digestion. PCR with primers binding to genomic *Actinoplanes* sp. SE50/110 DNA was used to verify complete removal of residual DNA. Quality and quantity of the RNA was analyzed with a NanoDrop 1000 spectrometer (Peqlab, Erlangen, Germany) and an Agilent RNA 6000 Pico kit run on an Agilent Bioanalyzer 2100 (Agilent Technologies, Santa Clara, CA, USA).

#### Whole genome oligonucleotide microarrays

Custom whole genome oligonucleotide microarrays representing the coding sequence of *Actinoplanes* sp. SE50/110 were designed with eArray (Agilent Technologies, Santa Clara, CA, USA) and ordered in the 4x44K format (Agilent Technologies, Santa Clara, CA, USA). These consist of 43,803 features representing 8238 genes and 1417 control spots. All experimental procedures, including sample preparation, cDNA synthesis and labeling, microarray hybridization and washing as well as scanning and feature extraction, were carried out as described by the manufacturer. The kit Two-Color Microarray-Based Prokaryote Analysis FairPlay III Labeling (Version 1.4, Agilent Technologies, Santa Clara, CA, USA) was used with the following adjustments, which were optimized and tested in previous experiments. The quantities and volumes of the components of the hybridization samples were adjusted to fit the 4x44K array format. The mix was prepared with 330 ng of each labeled cDNA and 11 μL gene expression blocking agent. The cDNA blocking mix was filled up to 55 μL with H_2_O and mixed with 55 μL Hi-RPM hybridization buffer. 100 μL of the hybridization mix were used for the hybridization of one array. Washing of the microarrays was carried out including stabilization and drying solution. The number and length of the washing steps was increased (two wash cycles, with 5 min wash buffer 1 and 1 min wash buffer 2) to reduce signal artifacts due to the high G + C content of *Actinoplanes* sp. SE50/110. Amersham CyDye mono-reactive dye packs were used from GE Healthcare (Little Chalfont, UK). All other microarray specific reagents as well as the hybridization oven and the microarray scanner were used from Agilent Technologies (Santa Clara, CA, USA).

Feature extraction was performed with the Agilent Feature Extraction Software Version 10.7.3.1 (Agilent Technologies, Santa Clara, CA, USA), applying the protocol GE2_107_Sep09. Subsequent data analysis, including LOWESS normalization and statistical analysis was performed with EMMA2 [60]. A *p*-value of 0.05 was used as a cut-off for significance and the M-value cut-offs for a false discovery rate of 0.01 were determined as 1.1 and −1.1, respectively.

#### Reverse transcription quantitative PCR

RT-qPCR was applied for relative mRNA quantification of single genes. Primers were designed to amplify 75 to 150 bps of intragenic regions (list of primers in Additional file 3). A SensiFast SYBR No-Rox One-Step Kit (Bioline, London, UK) and 96 well lightcycler plates (Sarstedt, Nümbrecht, Germany) were used for measurements in a LightCycler 96 System (Roche, Mannheim, Germany). 1 μL of template RNA, adjusted to 200 ng µL^-1^, was mixed with 19 μL master mix containing 1 μL of specific primers (10 μM each), 0.2 μL reverse transcriptase, 0.4 μL RNase inhibitor, 10 μL reaction mix and 7.4 μL 5 M betain. A minimum of three biological replicates in each technical duplicates was included for every measurement. Two negative controls with 1 μL H_2_O as template were included for each analyzed gene. Reverse transcription was performed at 45 °C for 20 min, followed by 2 min at 95 °C, a three step amplification (95 °C 5 s, 60 °C 10 s, 72 °C 10 s, 60 cycles) and a melting profile. The LightCycler 96 V1.1 software was used for inspection of control measurements and melting curve analysis. The relative RNA amount was normalized on total RNA (200 ng) and calculated as 2^-ΔCq^. ΔCq was calculated as the difference of the mean Cq in the mutant strain compared to the control strain.

### Heterologous expression and purification of AcrC in *Streptomyces lividans*

For the heterologous expression and purification of the AcrC protein, the *acrC* gene was cloned by Gibson assembly [59] into the multiple cloning site of the pGM1202 expression vector (G. Muth, unpubl. Data, available through Addgene # 69615) which includes a pSG5 origin of replication [61], the P_tipA_ promoter and a C-terminal His_6_-tag. The expression vector was transferred into *Streptomyces lividans* TK23 by conjugation. The strain was grown in 50 mL yeast extract-malt extract (YEME) medium with 50 μg mL^−1^ apramycin in a 250 mL flask at 28 °C and 180 rpm. After 3 days, 15 mL of the culture were transferred to 200 mL fresh YEME medium supplemented with 25 μg mL^−1^ thiostrepton to induce gene expression. The cells were cultivated for further 3 to 4 days at 28 °C and 180 rpm. Afterwards, the cells were harvested by centrifugation at 5000 *g* for 20 min at 4 °C. The pellet was resuspended in ice-cold lysis buffer (50 mM NaH_2_PO_4_, 300 mM NaCl, 10 mM imidazole, pH 8). Cell disruption was carried out with a French press for three times. Cell debris were separated from the soluble fraction by centrifugation (5000 *g*, 1 h) at 4 °C. The protein was purified from the supernatant using Protino® Ni-TED 1000 Packed Columns as described by the manufacturer (Macherey-Nagel, Düren, Germany) and stored in 30 mM Tris-HCl, 300 mM NaCl, pH 8.5 buffer at 4 °C.

### Electrophoretic mobility shift assays

DNA band shift assays were performed with Cy3-labeled PCR fragments and ds oligos for displacements (list of primers in Additional file 3). Cy3-labeled primers (Metabion, Steinkirchen, Germany) were used to produce PCR fragments, which were then purified by using a PCR Clean Up Kit (Macherey Nagel, Düren, Germany). The oligonucleotides were annealed by heating 5 min to 95 °C and then ramp to 4 °C at 0.1 °C s^−1^.

The binding assay was performed in a final reaction volume of 20 μL containing 80 pmol His-tagged AcrC protein, 4 μL of 5× EMSA binding buffer (100 mM Na_2_HPO_4_, 375 mM KCl, 25% Glycerin, pH 8), 2.5 mM MgCl_2_ and 0.1 mM EDTA. In addition, 0.05 μg of herring sperm DNA and 0.1 μg BSA (bovine serum albumin) was added to each reaction to block unspecific protein-DNA interactions. After incubation for 20 min at room temperature the samples were separated on a 10% native polyacrylamide gel (Biorad, Hercules, CA, USA) at 170 V using TBE (89 mM Tris base, 89 mM boric acid, 2 mM EDTA) as running buffer. The gel was scanned on a Typhoon 8600 Variable Mode Imager (GE Healthcare, Little Chalfont, UK).

## Additional files

Additional file 1: List of genes with significantly differential transcript abundancies in the mutant strain *ΔacrC* compared to the wild type in maltose and glucose minimal medium. (XLSX 122 kb)

Additional file 2: Relative RNA amounts of *malE* and *acbE* in the deletion strain compared to the wild type in different carbon sources. (PDF 206 kb)

Additional file 3: List of primers used in this study. (PDF 150 kb)

## Abbreviations

AcrC: Acarbose regulator C
ds oligo: Double-stranded oligonucleotides
DSE: Dyadic symmetry element
EMSA: Electrophoretic mobility shift assays
Glc-MM: Glucose minimal medium
HTH: Helix-turn-helix
Mal-MM: Maltose minimal medium
nAcb: Acarbose biosynthesis
RT-qPCR: Reverse transcription quantitative PCR
wt: Wild type

## References

[1] Creutzfeldt W (1988). Acarbose for the Treatment of Diabetes Mellitus.

[2] Bischoff H (1994). Pharmacology of α-glucosidase inhibition. Eur J Clin Investig.

[3] Wehmeier UF, Piepersberg W (2004). Biotechnology and molecular biology of the α-glucosidase inhibitor acarbose. Appl Microbiol Biotechnol.

[4] Wehmeier UF (2003). The biosynthesis and metabolism of Acarbose in *Actinoplanes* sp. SE 50/110: a progress report. Biocatal Biotransformation.

[5] Schwientek P, Szczepanowski R, Ruckert C, Kalinowski J, Klein A, Selber K (2012). The complete genome sequence of the acarbose producer *Actinoplanes* sp. SE50/110. BMC Genomics.

[6] Truscheit E, Frommer W, Junge B, Müller L, Schmidt DD, Wingender W (1981). Chemistry and biochemistry of microbial α-Glucosidase inhibitors. Angew Chem Int Ed Engl.

[7] Parenti F, Coronelli C (1979). Members of the genus *Actinoplanes* and their antibiotics. Annu Rev Microbiol.

[8] Vobis G, Schäfer J, Kämpfer P, Bergey DH, Whitman WB, Goodfellow M, Kämpfer P, Busse H-J (2012). Actinoplanes. Bergey’s manual of systematic bacteriology.

[9] Zhang C-S, Stratmann A, Block O, Brückner R, Podeschwa M, Altenbach H-J (2002). Biosynthesis of the C(7)-cyclitol moiety of acarbose in *Actinoplanes* species SE50/110. 7-O-phosphorylation of the initial cyclitol precursor leads to proposal of a new biosynthetic pathway. J Biol Chem.

[10] Wendler S, Hurtgen D, Kalinowski J, Klein A, Niehaus K, Schulte F (2013). The cytosolic and extracellular proteomes of *Actinoplanes* sp. SE50/110 led to the identification of gene products involved in acarbose metabolism. J Biotechnol.

[11] Wendler S, Otto A, Ortseifen V, Bonn F, Neshat A, Schneiker-Bekel S (2016). Comparative proteome analysis of *Actinoplanes* sp. SE50/110 grown with maltose or glucose shows minor differences for acarbose biosynthesis proteins but major differences for saccharide transporters. J Proteome.

[12] Gren T, Ortseifen V, Wibberg D, Schneiker-Bekel S, Bednarz H, Niehaus K (2016). Genetic engineering in *Actinoplanes* sp. SE50/110 - development of an intergeneric conjugation system for the introduction of actinophage-based integrative vectors. J Biotechnol.

[13] Wolf T, Gren T, Thieme E, Wibberg D, Zemke T, Puhler A, Kalinowski J (2016). Targeted genome editing in the rare actinomycete *Actinoplanes* sp. SE50/110 by using the CRISPR/Cas9 system. J Biotechnol.

[14] Wolf T, Schneiker-Bekel S, Neshat A, Ortseifen V, Wibberg D, Zemke T (2017). Genome improvement of the acarbose producer *Actinoplanes* sp. SE50/110 and annotation refinement based on RNA-seq analysis. J Biotechnol.

[15] Rockser Y, Wehmeier UF (2009). The gac-gene cluster for the production of acarbose from *Streptomyces glaucescens* GLA.O—identification, isolation and characterization. J Biotechnol.

[16] Guo X, Geng P, Bai F, Bai G, Sun T, Li X (2012). Draft genome sequence of *Streptomyces coelicoflavus* ZG0656 reveals the putative biosynthetic gene cluster of acarviostatin family alpha-amylase inhibitors. Lett Appl Microbiol.

[17] Virolle M-J, Long CM, Shing C, Bibb MJ (1988). Cloning, characterisation and regulation of an α-amylase gene from *Streptomyces venezuelae*. Gene.

[18] Virolle MJ, Gagnat J (1994). Sequences involved in growth-phase-dependent expression and glucose repression of a *Streptomyces* α-amylase gene. Microbiology.

[19] Ortseifen V, Winkler A, Albersmeier A, Wendler S, Puhler A, Kalinowski J, Ruckert C (2015). Complete genome sequence of the actinobacterium *Streptomyces glaucescens* GLA.O (DSM 40922) consisting of a linear chromosome and one linear plasmid. J Biotechnol.

[20] Altschul S (1997). Gapped BLAST and PSI-BLAST: a new generation of protein database search programs. Nucleic Acids Res.

[21] Edgar RC (2004). MUSCLE: multiple sequence alignment with high accuracy and high throughput. Nucleic Acids Res.

[22] van Wezel GP, White J, Young P, Postma PW, Bibb MJ (1997). Substrate induction and glucose repression of maltose utilization by *Streptomyces coelicolor* A3(2) is controlled by malR, a member of the lacI-galR family of regulatory genes. Mol Microbiol.

[23] Schlösser A, Weber A, Schrempf H (2001). Synthesis of the *Streptomyces lividans* maltodextrin ABC transporter depends on the presence of the regulator MalR. FEMS Microbiol Lett.

[24] Gust B, Challis GL, Fowler K, Kieser T, Chater KF (2003). PCR-targeted *Streptomyces* gene replacement identifies a protein domain needed for biosynthesis of the sesquiterpene soil odor geosmin. Proc Natl Acad Sci U S A.

[25] Gust B, Kieser T, Chater K. REDIRECT technology: PCR targeting system in *Streptomyces coelicolor* A3(2) 2002. Norwich: John Innes Centre.

[26] Wendler S, Ortseifen V, Persicke M, Klein A, Neshat A, Niehaus K (2014). Carbon source dependent biosynthesis of acarviose metabolites in *Actinoplanes* sp. SE50/110. J Biotechnol.

[27] Wendler S, Otto A, Ortseifen V, Bonn F, Neshat A, Schneiker-Bekel S (2015). Comprehensive proteome analysis of *Actinoplanes* sp. SE50/110 highlighting the location of proteins encoded by the acarbose and the pyochelin biosynthesis gene cluster. J Proteome.

[28] Rhodes D, Schwabe JW, Chapman L, Fairall L (1996). Towards an understanding of protein-DNA recognition. Philos Trans R Soc Lond Ser B Biol Sci.

[29] Huffman JL, Brennan RG (2002). Prokaryotic transcription regulators: more than just the helix-turn-helix motif. Curr Opin Struct Biol.

[30] Weickert MJ, Adhya S (1992). A family of bacterial regulators homologous to gal and Lac repressors. J Biol Chem.

[31] Swint-Kruse L, Matthews KS (2009). Allostery in the LacI/GalR family: variations on a theme. Curr Opin Microbiol.

[32] Herrmann S, Siegl T, Luzhetska M, Petzke L, Jilg C, Welle E (2012). Site-specific recombination strategies for engineering actinomycete genomes. Appl Environ Microbiol.

[33] Myronovskyi M, Luzhetskyy A (2013). Genome engineering in actinomycetes using site-specific recombinases. Appl Microbiol Biotechnol.

[34] Fedoryshyn M, Petzke L, Welle E, Bechthold A, Luzhetskyy A (2008). Marker removal from actinomycetes genome using Flp recombinase. Gene.

[35] Zelyas N, Tahlan K, Jensen SE (2009). Use of the native *flp* gene to generate in-frame unmarked mutations in *Streptomyces* spp. Gene.

[36] Mortazavi A, Williams BA, McCue K, Schaeffer L, Wold B (2008). Mapping and quantifying mammalian transcriptomes by RNA-Seq. Nat Methods.

[37] Wang Z, Gerstein M, Snyder M (2009). RNA-Seq: a revolutionary tool for transcriptomics. Nat Rev Genet.

[38] van Wezel GP, White J, Bibb MJ, Postma PW (1997). The malEFG gene cluster of *Streptomyces coelicolor* A3(2): characterization, disruption and transcriptional analysis. Mol Gen Genet.

[39] Nguyen J, Francou F, Virolle MJ, Guérineau M (1997). Amylase and chitinase genes in *Streptomyces lividans* are regulated by reg1, a pleiotropic regulatory gene. J Bacteriol.

[40] Nguyen J (1999). The regulatory protein Reg1 of *Streptomyces lividans* binds the promoter region of several genes repressed by glucose. FEMS Microbiol Lett.

[41] Virolle M-J, Bibb MJ (1988). Cloning, characterization and regulation of an α-amylase gene from *Streptomyces limosus*. Mol Microbiol.

[42] Henkin TM, Grundy FJ, Nicholson WL, Chambliss GH (1991). Catabolite repression of α-amylase gene expression in *Bacillus subtilis* involves a trans-acting gene product homologous to the *Escherichia coli lacl* and *galR* repressors. Mol Microbiol.

[43] Afzal M, Shafeeq S, Manzoor I, Kuipers OP (2015). Maltose-dependent transcriptional regulation of the mal Regulon by MalR in *Streptococcus pneumoniae*. PLoS One.

[44] Wehmeier UF, Piepersberg W (2009). Chapter 19 Enzymology of Aminoglycoside Biosynthesis—Deduction from Gene Clusters. Complex Enzymes in Microbial Natural Product Biosynthesis, Part B: Polyketides, Aminocoumarins and Carbohydrates: Elsevier.

[45] Hemker M, Stratmann A, Goeke K, Schroder W, Lenz J, Piepersberg W, Pape H (2001). Identification, cloning, expression, and characterization of the extracellular acarbose-modifying glycosyltransferase, AcbD, from Actinoplanes sp. strain SE50. J Bacteriol.

[46] Leemhuis H, Wehmeier UF, Dijkhuizen L (2004). Single amino acid mutations interchange the reaction specificities of Cyclodextrin Glycosyltransferase and the Acarbose-modifying enzyme Acarviosyl Transferase. Biochemistry.

[47] Lewis M, Chang G, Horton NC, Kercher MA, Pace HC, Schumacher MA (1996). Crystal structure of the lactose Operon repressor and its complexes with DNA and inducer. Science.

[48] Matthews KS, Nichols JC (1998). Lactose Repressor Protein: Functional Properties and Structure. Progress in nucleic acid research and molecular biology.

[49] Oehler S, Eismann ER, Kramer H, Muller-Hill B (1990). The three operators of the *lac* operon cooperate in repression. EMBO J.

[50] Wong OK, Guthold M, Erie DA, Gelles J (2008). Interconvertible lac repressor-DNA loops revealed by single-molecule experiments. PLoS Biol.

[51] Rutkauskas D, Zhan H, Matthews KS, Pavone FS, Vanzi F (2009). Tetramer opening in LacI-mediated DNA looping. Proc Natl Acad Sci U S A.

[52] Boos W, Shuman H (1998). Maltose/maltodextrin system of *Escherichia coli*: transport, metabolism, and regulation. Microbiol Mol Biol Rev.

[53] Seibold GM, Wurst M, Eikmanns BJ (2009). Roles of maltodextrin and glycogen phosphorylases in maltose utilization and glycogen metabolism in *Corynebacterium glutamicum*. Microbiology.

[54] Brunkhorst C, Schneider E (2005). Characterization of maltose and maltotriose transport in the acarbose-producing bacterium *Actinoplanes* sp. Res Microbiol.

[55] Grant SG, Jessee J, Bloom FR, Hanahan D (1990). Differential plasmid rescue from transgenic mouse DNAs into *Escherichia coli* methylation-restriction mutants. Proc Natl Acad Sci U S A.

[56] Kieser T, Bibb MJ, Buttner MJ, Chater KF, Hopwood DA (2000). Practical *Streptomyces* genetics.

[57] Hopwood DA, Hintermann G, Kieser T, Wright HM (1984). Integrated DNA sequences in three streptomycetes form related autonomous plasmids after transfer to *Streptomyces lividans*. Plasmid.

[58] Bierman M, Logan R, O’Brien K, Seno ET, Nagaraja Rao R, Schoner BE (1992). Plasmid cloning vectors for the conjugal transfer of DNA from *Escherichia coli* to *Streptomyces* spp. Gene.

[59] Gibson DG, Young L, Chuang R-Y, Venter JC, Hutchison CA, Smith HO (2009). Enzymatic assembly of DNA molecules up to several hundred kilobases. Nat Methods.

[60] Dondrup M, Albaum SP, Griebel T, Henckel K, Junemann S, Kahlke T (2009). EMMA 2--a MAGE-compliant system for the collaborative analysis and integration of microarray data. BMC Bioinformatics.

[61] Muth G, Nußbaumer B, Wohlleben W, Pühler A (1989). A vector system with temperature-sensitive replication for gene disruption and mutational cloning in streptomycetes. Mol Gen Genet.

[62] Finn RD, Coggill P, Eberhardt RY, Eddy SR, Mistry J, Mitchell AL (2016). The Pfam protein families database: towards a more sustainable future. Nucleic Acids Res.

[63] Kearse M, Moir R, Wilson A, Stones-Havas S, Cheung M, Sturrock S (2012). Geneious basic: an integrated and extendable desktop software platform for the organization and analysis of sequence data. Bioinformatics.

