# Supplementary material for: The MalR type regulator AcrC is a transcriptional repressor of acarbose biosynthetic genes in Actinoplanes sp. SE50/110
Source: BMC Genomics. 2017 Jul 25;18:562. doi: 10.1186/s12864-017-3941-x (PMC5526262; doi:10.1186/s12864-017-3941-x)
Supplement: Supplementary file 2 — Relative RNA amounts of malE and acbE in the deletion strain compared to the wild type in different carbon sources. (PDF 206 kb) [file 12864_2017_3941_MOESM2_ESM.pdf]

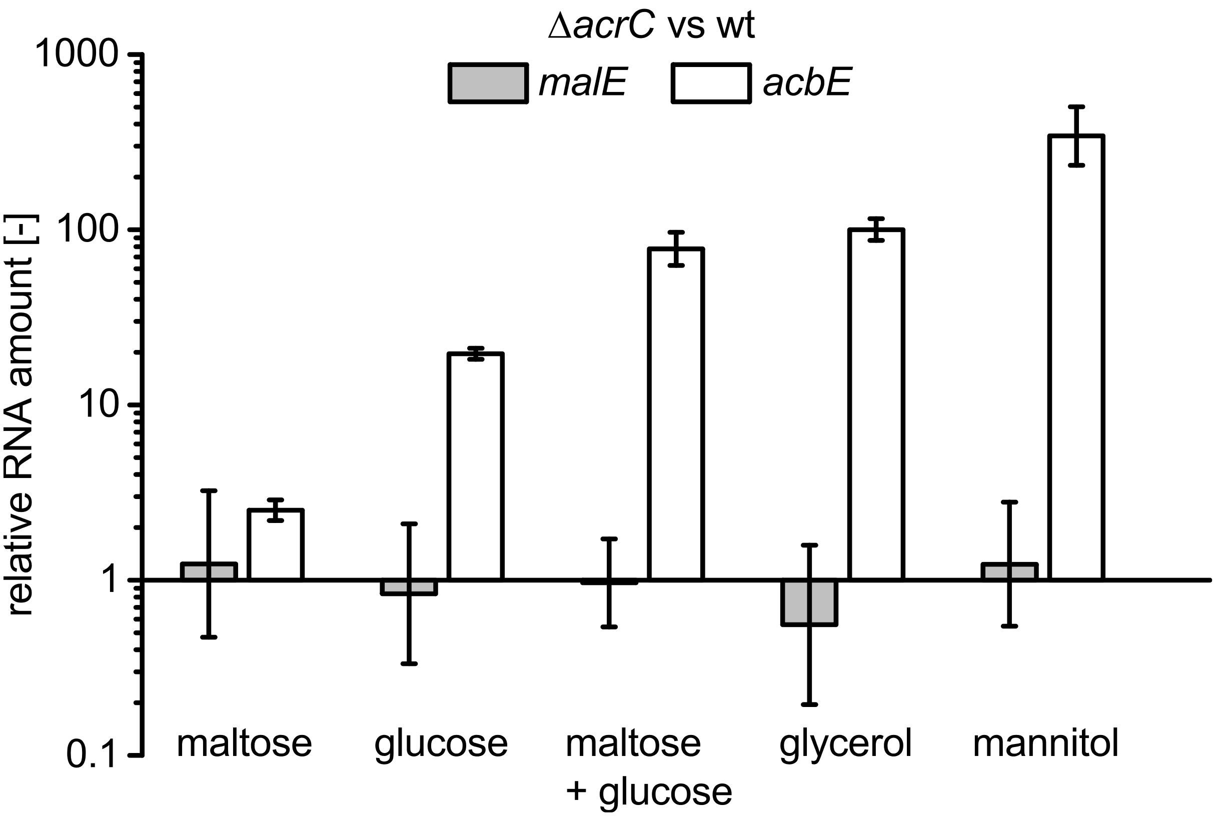

Relative RNA amounts of *malE* and *acbE* in the deletion strain compared to the wild type in different carbon sources. Relative transcript abundances measured by RT-qPCR of the deletion strain *Actinoplanes* sp. SE50/110 Δ*acrC* compared with the wild type *Actinoplanes* sp. SE50/110 (wt). RNA was isolated from the growth phase of shake flask cultivations of both strains in minimal medium supplemented with 2.4 C-mole of the carbon sources indicated in the figure. The means and standard derivations of three biological replicates are shown.
