# Supplementary material for: The MalR type regulator AcrC is a transcriptional repressor of acarbose biosynthetic genes in Actinoplanes sp. SE50/110
Source: BMC Genomics. 2017 Jul 25;18:562. doi: 10.1186/s12864-017-3941-x (PMC5526262; doi:10.1186/s12864-017-3941-x)
Supplement: Supplementary file 3 — List of primers used in this study. (PDF 150 kb) [file 12864_2017_3941_MOESM3_ESM.pdf]

Table S1: List of primers used in this study.

Name	Sequence 5' – 3'	Description
acrC_redirect_fwd	ACGGCTCGGCACTACAGTGACAACATGCGTGCACGGATGATTCCGGGGATCCGTCGACC	PCR targeting of *acrC*
acrC_redirect_rev	GCAAGAAGTTGCAGCAAGTTTCCCGGGCGGTGCGGGTCATGTAGGCTGGAGCTGCTTC	
hph_fwd	GAGTTATCGAGATTTTCAGGAGCTAAGGAAGCTAAAATGCCCGTAGAGATTGGCGATCCC	Red/ET exchange of chloramphenicol resistance cassette with hygromycin resistance cassette on pcc2FOS
hph_rev	AGGCGTTTAAGGGCACCAATAACTGCCTTAAAAAAATTACAGGCGCCGGGGGCGGTGTC	
acrC_seq_fwd	TGTCTTGGGCCCGGACATAG	Verification of *acrC* deletion
acrC_seq_rev	GCGGATTCTTGCCAGGGATG	
fwd_pSET152_malR	CGACGCCTCGGCTGCCTGAGTCATAGCTGTTTCCTGTGTG	Amplification of backbone for Gibson assembly of *acrC* in pSET152
rev_pSET152_malR	GCGAGCATGCTCTGCCTGGTCGATCTTTGTAGAAACCATC	
fwd_malR_pSET152	CGCCGATGGTTTCTACAAAGATCGACCAGGCAGAGCATGC	Amplification of *acrC* for Gibson assembly of *acrC* in pSET152
rev_malR_pSET152	ACAGGAAACAGCTATGACTCAGGCAGCCGAGGCGTCGGCG	
fwd_pSET152_hph	ACACCGCCCCCGGCGCCTGAGCTCATGAGCGGAGAACGAG	Amplification of backbone for Gibson assembly of *hph* in pGM1202
rev_pSET152_hph	CAGGGATTCTTGTGTCACGACATTGCACTCCACCGCTGAT	
fwd_hph_pSET152	GCGGTGGAGTGCAATGTCGTGACACAAGAATCCCTGTTAC	Amplification of *hph* for Gibson assembly of *hph* in pSET152
rev_hph_pSET152	TCATCTCGTTCTCCGCTCATGAGCTCAGGCGCCGGGGGCG	

fwd_pGM1202_acrC	CCGACGCCTCGGCTGCCGGATCCCATCATCATCATCATCA	Amplification of backbone for Gibson assembly of *acrC* in pGM1202
rev_pGM1202_acrC	TTGACGGGCGATGTCCGCCATATGTCCGCTCCCTTCTCTG	
fwd_acrC_pGM1202	TCAGAGAAGGGAGCGGACATATGGCGGACATCGCCCGTCA	Amplification of *acrC* for Gibson assembly of *acrC* in pGM1202
rev_acrC_pGM1202	AATGATGATGATGATGATGGGATCCGGCAGCCGAGGCGTC	
fwd_RT-qPCR_acrC	GAGCGACTGCTCAACAAG	RT-qPCR of *acrC*
rev_RT-qPCR_acrC	GTCGTCGAAACCGATCAC	
fwd_RT-qPCR_malE	GGAATGGCGTTCTTACCG	RT-qPCR of *malE*
rev_RT-qPCR_malE	CGAAGGGCAAGAACAAGG	
fwd_RT-qPCR_acbE	CGAGCAGCTCTACCTGGTGATGC	RT-qPCR of *acbE*
rev_RT-qPCR_acbE	GCTTGTTCTTGAAGATCGGCGTCAG	
fwd_RT-qPCR_acbD	GCCAGCAACATCAAAGTG	RT-qPCR of *acbD*
rev_RT-qPCR_acbD	TGGTAGCGATCGTTGAAG	
fwd_acbE_acbD	Cy3-CATCCATCGTGGACCCTCTC	EMSA of intergenic region acbE/acbD
rev_acbE_acbD	GACGTTGCACGAGCTTCTCC	
fwd_malE_acrC	Cy3-TGCGCATGAAAATACTCCCG	EMSA of intergenic region malE/acrC
rev_malE_acrC	GGCGATGTCCGCCATCCGTG	
fwd_dapE2_6389	Cy3-ACGTCGCCGGTGAGATCAAG	EMSA of intergenic region dapE2/ACSP50_6389
rev_dapE2_6389	ACTGGACGCTGACGCTCAAC	
fwd_acrC_site_acbE	AAGAACTTGCTGTTTTAGCAAGAAGTT	Displacement oligo 1
rev_acrC_site_acbE	AACTTCTTGCTAAAACAGCAAGTTCTT	
fwd_acrC_site_acbD	GTCTTCTGCAAGTTCTTGCAGCGGTC	Displacement oligo 2
rev_acrC_site_acbD	GACCGCTGCAAGAACTTGCAGAAGAC	
fwd_acrC_site_acrC	AAGCTCTTGCAGTCAATGGAAGGGCTT	Displacement oligo 3
rev_acrC_site_acrC	AAGCCCTTCCATTGACTGCAAGAGCTT	
